# AIVariant: a deep learning-based somatic variant detector for highly contaminated tumor samples

**DOI:** 10.1038/s12276-023-01049-2

**Published:** 2023-08-01

**Authors:** Hyeonseong Jeon, Junhak Ahn, Byunggook Na, Soona Hong, Lee Sael, Sun Kim, Sungroh Yoon, Daehyun Baek

**Affiliations:** 1 Interdisciplinary Program in Bioinformatics, Seoul National University, Seoul, 08826 Republic of Korea; 2 Genome4me Inc., Seoul, 08826 Republic of Korea; 3 School of Biological Sciences, Seoul National University, Seoul, 08826 Republic of Korea; 4 Department of Electrical and Computer Engineering, Seoul National University, Seoul, 08826 Republic of Korea; 5 AIGENDRUG Co., Ltd., Seoul, 08826 Republic of Korea; 6 Department of Software and Computer Engineering, Ajou University, Suwon, 16499 Republic of Korea; 7 Department of Computer Science and Engineering, Seoul National University, Seoul, 08826 Republic of Korea; 8 Interdisciplinary Program in Artificial Intelligence, Seoul National University, Seoul, 08826 Republic of Korea

**Keywords:** Machine learning, Cancer, Data processing

## Abstract

The detection of somatic DNA variants in tumor samples with low tumor purity or sequencing depth remains a daunting challenge despite numerous attempts to address this problem. In this study, we constructed a substantially extended set of actual positive variants originating from a wide range of tumor purities and sequencing depths, as well as actual negative variants derived from sequencer-specific sequencing errors. A deep learning model named AIVariant, trained on this extended dataset, outperforms previously reported methods when tested under various tumor purities and sequencing depths, especially low tumor purity and sequencing depth.

## Cancer: Detecting genetic variants with AI

An artificial intelligence (AI) system trained to detect genetic variants in tumor cell DNA sequence data out-performs existing methods, especially for impure samples contaminated with healthy cells. Identifying the DNA sequence variants in cancer cells is essential for understanding the genetic basis of specific cancers. One major obstacle has been the low proportion of tumor cells in many clinical samples and the challenge of obtaining sequence data specific to the cancer cells. This is especially problematic with some common types of cancer, including those affecting the lungs, kidneys, head, neck and pancreas. Researchers in South Korea led by Daehyun Baek at Seoul National University developed the ‘AIVariant’ method to address these difficulties. The high precision of AIVariant analysis significantly advances the state-of-the-art, which should benefit cancer research and diagnosis, and the search for better treatments.

## Introduction

Somatic DNA variants are the primary cause of cancer biogenesis and progression. Accurate detection of somatic variants within a cancer genome is essential for understanding the comprehensive landscape of cancer genetics and the fundamental basis for cancer diagnosis and treatment. Despite its significance, the detection of somatic variants remains challenging for the following reasons. One of the main obstacles is tumor purity, which refers to the proportion of tumor cells in an obtained tumor sample. The presence of normal cells such as stromal and immune cells within the tumor microenvironment can make it difficult to collect a pure sample of tumor cells^1–3^. Previous studies have reported that tumor tissue samples often contain normal cells and exhibit various tumor purities^4–7^. Samples with low tumor purity generally have a low proportion of sequencing reads supporting true somatic variants, making the detection of these variants difficult. Accordingly, a variant detector that can accurately detect variants with low tumor purities would greatly facilitate cancer diagnosis and treatment. This is especially important for cancer types, such as lung squamous-, kidney renal clear-, and head and neck squamous-cell carcinoma, as well as lung and pancreatic adenocarcinoma^5,6^, where low tumor purity samples are frequently discovered. Similar to low tumor purity, a low sequencing depth results in a small number of reads supporting true somatic variants and thus makes somatic variant detection more difficult. Despite advances in next-generation sequencing technology, whole genome sequencing (WGS) of a tumor sample with a higher sequencing depth often leads to an inhibitory sequencing cost^8^. Another challenge stems from sequencing errors introduced by distinct biases specific to a particular sequencer. For example, the widely used Illumina sequencers are known to have various sequencer-specific errors. It has been reported that higher sequencing error rates are observed near certain sequence motifs^9,10^, homopolymers^11^, and read ends^10,12^. Bases with similar fluorophore emission spectra can be miscalled^10^. Sequencing errors have been found to accumulate in a specific strand direction^12^, and reverse reads tend to be more error-prone than forward reads in paired-end sequencing^13^. Furthermore, low confidence in Illumina short-read sequencing in low-complexity regions, such as tandem repeats, has been reported^14^. Collectively, these sequencer-specific errors may be misinterpreted as true somatic variants, resulting in numerous false positives if not properly addressed. A large number of methods have been developed to detect somatic variants from sequencing data, including Mutect2^15^, Strelka2^16^, MuSE^17^, VarScan2^18^, SomaticSniper^19^, and NeuSomatic^20,21^. However, while these methods accurately detect somatic variants with high tumor purity and sequencing depth, all of these methods suffer from low accuracy for low tumor purity or sequencing depth. Moreover, most of these methods do not fully address sequencer-specific sequencing errors, although some employ naïve heuristics to address this issue. In this study, we developed a new somatic variant detector, AIVariant, by training a deep learning model with an extended set of somatic variants originating from a wide range of tumor purities, sequencing depths, and sequencer-specific sequencing errors. We demonstrated that systematically constructing a comprehensive dataset that encompasses actual positive and negative variants is crucial for the development of an accurate somatic variant detector. AIVariant exhibits both high precision and sensitivity for somatic variant detection, especially for low tumor purity and sequencing depth.

## Materials and methods

### Somatic variant detection

The detection process consisted of four key steps: preprocessing, candidate search, image generation, and somatic variant calling. During preprocessing, low-quality sequencing reads of tumor/normal binary alignment MAP (BAM) files were filtered to exclude erroneous sequencing results. Subsequently, normalized base quality scores were calculated for the base quality scores of the remaining reads. The candidate search step identified probable somatic variant candidates by scanning tumor/normal paired sequencing data. These candidates were identified based on the base qualities of the variant alleles, the number of reads aligned with the position of interest, and a Bayesian classifier score. In the image generation step, two types of input data matrices were generated for each candidate: the first data matrix, termed an AIVariant image, represents raw alignment features of up to 100 sequencing reads around a variant, including nucleotide compositions, qualities of alignments, and epigenetic features. The second data matrix, termed an AIVaraint stat image, represents statistically summarized alignment features of all the aligned reads around a variant (Supplementary Fig. 2a). In the somatic variant calling step, a deep neural network was employed to calculate the posterior probability for a somatic variant at the candidate positions with the generated images and Bayesian classifier scores. For a detailed description of each step, see Supplementary Materials and Methods.

### Generation of simulated whole genome sequencing data with an extended set of variants (eWGS)

eWGS data were generated by using Illumina WGS datasets from three sources. The FASTQ files of the NA12877 200x WGS data^22^ (https://www.ebi.ac.uk/ena/browser/view/PRJEB3246) and CHM1/CHM13 WGS data^23^ (https://www.ebi.ac.uk/ena/browser/view/PRJEB13208) were obtained from the European Nucleotide Archive (ENA), while the 81 WGS tumor BAM files from 78 patients with cancer were obtained from the International Cancer Genome Consortium (ICGC)^24^ (https://dcc.icgc.org/repositories). Aligned reads of the BAM files were extracted to generate FASTQ files. To match the different sequencing read lengths of WGS data to a uniform read length, the reads of the FASTQ files were trimmed from the 3’ end to a length of 100 bp. The trimmed reads were then aligned to the reference genome using ‘BWA MEM (v0.7.8)’ with default options. From aligned reads, the filtering process removed low-quality reads: unmapped reads, PCR or optically duplicated reads, reads with secondary alignments, and reads with a mapping quality of 0. The generated BAM files from the previous steps were then downsampled to a sequencing depth (SD) of 30. For NA12877, the BAM file was split into three BAM files with no overlapping sequencing reads, each of which was downsampled to an SD of 30, resulting in normal, tumor backbone, and normal simulation WGS data. Actual positive (AP) and actual negative (AN) somatic single nucleotide variants (SNVs) were spiked into the tumor backbone WGS data to generate tumor WGS data. This process utilized WGS data from patients with cancer and CHM1/CHM13. First, the reads overlapping the ±50 nt flanking regions of the spiked-in SNVs were removed from the tumor backbone WGS BAM file. Second, reads overlapping the identical ±50 nt flanking regions were extracted from the preprocessed BAM files of patients with cancer or CHM1/CHM13. Finally, the extracted reads were spiked into the tumor backbone WGS data. ICGC tumor WGS data with a tumor purity of nearly 100% were identified based on the distribution of variant allele frequencies (VAFs) for somatic SNVs. More specifically, tumor WGS data with median VAFs for somatic SNVs between 0.4 and 0.6 were selected (although the theoretical median VAF of tumor tissue samples with 100% tumor purity (TP) is 0.5 and the distribution of VAF for a 100% TP tumor tissue sample has two peaks at 0.5 and 1.0 with a much higher peak for 0.5, several factors such as low TP or SD and subclonality can affect the ideal distribution of VAFs and result in a slightly different median VAF value). We identified 9 ICGC WGS datasets with almost 100% TP from each of 9 ICGC projects (each project accounted for a specific cancer type): PACA-AU (pancreatic cancer), PAEN-AU (pancreatic cancer endocrine neoplasms), PBCA-DE (pediatric brain cancer), LIRI-JP (liver cancer), OV-AU (ovarian cancer), PRAD-UK (prostate adenocarcinoma), THCA-US (head and neck thyroid carcinoma), MALY-DE (malignant lymphoma), and MELA-AU (skin cancer). To simulate various TPs in the tumor WGS data, the initial tumor WGS data with almost 100% TP and normal simulation WGS BAM files were downsampled using the ‘Samtools view (v1.9)’ in a manner that reflected the target TP, N%. For example, to generate a tumor WGS dataset with 40% TP, 40% of the reads from the tumor WGS data and 60% of the reads from the normal simulation WGS data were downsampled and merged using ‘Picard MergeSamFiles (v2.18.1)’. Note that during the simulations, the value of SD did not change, so if we began with data showing an SD of 30, the simulation resulted in data with the desired TP and an SD of 30. To simulate various SDs, the BAM files for normal WGS data with an SD of 30 and tumor WGS data with an SD of 30 were downsampled to target SDs of 25, 20, and 15, for example, by downsampling 50% of the reads from the BAM file with an SD of 30 to generate an SD of 15. The resulting eWGS dataset, which includes both normal and tumor WGS data with nine different TPs at four different SDs, was further split into two datasets: the training eWGS and test eWGS datasets. The training eWGS dataset consisted of sequencing reads aligned to odd-numbered chromosomes (chr1, 3, 5,…, and 21), whereas the test eWGS dataset consisted of sequencing reads aligned to even-numbered chromosomes (chr2, 4, 6,…, and 22).

### Identification of actual positive variants

The AP variants were identified using somatic SNVs in ICGC WGS data of 9 different cancer types and germline SNVs in CHM1/CHM13 WGS data^23^. For ICGC WGS data, we downloaded Variant Call Format (VCF) files that include the list of somatic SNVs identified by ICGC projects from the ICGC Data Portal (https://dcc.icgc.org/repositories). We used only somatic SNVs that had been consensually called by all four somatic variant detectors employed in the ICGC project. For the AP variants, which should include only highly probable somatic SNVs, we further filtered these variants based on base qualities and the number of sequencing reads aligned at a variant position, a filtering process referred to as the candidate search (see Supplementary Materials and Methods). For the CHM1/CHM13 WGS data, we downloaded relevant VCF files that included two germline SNV lists for WGS data generated by Illumina and Pacific Bioscience (PacBio) sequencers (https://github.com/lh3/CHM-eval/release). To obtain the AP variants in the CHM1/CHM13 WGS data, we collected only the germline variants supported by both sequencers and applied the aforementioned filtering process for the ICGC WGS data to these variants (see Supplementary Materials and Methods).

### Identification of actual negative variants

AN SNVs derived from Illumina sequencer-specific errors were collected by comparing two CHM1/CHM13 WGS datasets generated by the Illumina and PacBio platforms. PacBio sequencer is based on long-read sequencing and is useful for complementing the limitations of the short-read sequencing of Illumina sequencer^23^. We identified highly probable somatic variants in the Illumina-generated WGS data based on base qualities and the number of sequencing reads aligned at a variant, termed the candidate search (see Supplementary Materials and Methods). Among these variants, we selected variants that were not called as germline variants both in the Illumina-generated WGS data and the PacBio-generated WGS data based on downloaded VCF files for CHM1/CHM13 (see section “Identification of actual positive variants”). These chosen variants were spiked into the tumor backbone WGS data to simulate AN variants derived from Illumina sequencer-specific errors. Another type of AN variant reflects the false positive somatic variants derived from the random nature of the tumor and normal tissue sampling and sequencing. More specifically, when both tumor and normal tissues have a germline variant or sequencing errors at a certain genomic position, sampling and sequencing can result in the sequencing data in which the tumor WGS holds a higher number of reads that include the variant than the normal WGS data, and this position can be falsely identified as a somatic variant. This type of AN variant was simulated during eWGS data generation. For certain positions at which tumor eWGS data were not spiked in with any AP variants or AN variants derived from Illumina sequencer-specific errors, the sequencing reads from the high-coverage normal WGS data (200x NA12877 WGS data) were randomly shuffled and divided into two groups to conform the reads aligned to these positions in the tumor eWGS and normal eWGS datasets, respectively. Due to the random nature of shuffling and division, the tumor eWGS data may include a higher number of sequencing reads with alternate alleles than the normal eWGS data at some positions. While this creates alignments around these positions that resemble somatic variants, these positions cannot be considered true somatic variants since the aligned reads for both the tumor eWGS and normal eWGS data were originally extracted from the same normal WGS data with high sequencing depth. Therefore, these positions likely represent sequencing errors or germline variants and are considered AN variants.

### Measuring the accuracy of somatic variant detection

To evaluate the accuracy of the somatic variant detection methods, we calculated the area under the precision-recall (PR) curve (PR-AUC). For a fair comparison, PR curves were extrapolated with a linear line toward a precision of 0.0 and recall of 1.0 point (see Supplementary Materials and Methods) before calculating PR-AUC. For the accuracy evaluation of other somatic variant detectors, we used the default settings of Mutect2^15^ (v4.1.8.1), Strelka2^16^ (v2.9.10), NeuSomatic^21^ (v0.1.4), MuSE^17^ (v1.0rc), VarScan2^18^ (v2.4.4), and SomaticSniper^19^ (v1.0.5.0). For NeuSomatic, we used ‘NeuSomatic_v0.1.4_standalone_SEQC-WGS-GT50-SpikeWGS10.pth' (https://github.com/bioinform/neusomatic) as the model. For accuracy evaluation, we utilized 34 TP and SD cases with nine TPs (20–100% with a step size of 10%) and four SDs (30, 25, 20, and 15x). TPs of 20% and 30% at an SD of 15 were excluded from the accuracy evaluation because of insufficient variant-supporting reads for a significant proportion of AP variants. To assess the test eWGS data for a specific cancer tissue type, we masked AP variant positions from the evaluation of the other eight cancer tissue types. For the evaluation of pancreatic cancer, we used AP variants from two ICGC projects, PACA-AU and PAEN-AU.

### Public somatic variant benchmark dataset

We obtained the ICGC-TCGA DREAM Somatic Variant Calling Challenge Synthetic Data3^25^ BAM files (https://www.ncbi.nlm.nih.gov/sra/SRX1026041) and truth somatic variant VCF files (https://www.synapse.org/#!Synapse:syn2177211). Read trimming, read alignment, low-quality read filtering, downsampling to an SD of 30, and simulation of various TPs and SDs were performed on the extracted reads from the two downloaded BAM files in a manner identical to that for the eWGS data (see Generation of simulated WGS data with an extended set of variants). The 6391 AP variant positions were obtained from the SNV positions in the downloaded truth somatic variant VCF files. We obtained 50x NA12877 and NA12878 BAM files^22^ (https://www.ebi.ac.uk/ena/browser/view/PRJEB3381) along with Platinum variant call VCF files (https://www.ebi.ac.uk/ena/browser/view/PRJEB8596). The preprocessing, 30x downsampling, and simulation of various TPs and SDs were performed identically to the construction of the DREAM-Challenge dataset. AP variants were simulated by considering the NA12878 BAM file as tumor sequencing data and the NA12877 BAM file as normal sequencing data, identical to the procedure in previous studies^16,20^. Specifically, the AP variants were derived from germline SNV positions of NA12878, which are non-germline variant positions in NA12877. The accuracy evaluation for somatic variant detection was restricted to the chr1 confidence regions of NA12877 and NA12878 with 92,748 AP variants.

### Training models to analyze the impact of the extended dataset of actual positive and negative variants

Three analyses were designed to assess the impact of three factors that were simulated on the extended set of variants: (1) various TPs, (2) various SDs, and (3) sequencer-specific sequencing errors. For each analysis, we trained two models with the same architecture: the extended and baseline models. In the analysis regarding various TPs, we trained a model on 9 different TPs ranging from 20–100% with a step size of 10% to create the extended model. To compare the accuracies, the baseline model was trained on a 100% TP. Both training datasets for the two models were matched to a size of 49,000 AP and AN variants through downsampling for a fair comparison. Similarly, in the analysis of the impact of various sequencing depths, the extended model was trained on four different SDs, 15, 20, 25, and 30, while the baseline model was trained on only an SD of 30. The training datasets were downsampled to 33,500 samples each. For sequencer-specific sequencing errors, the baseline model was trained on a subset of AP and AN variants that were not derived from sequencer-specific sequencing errors. In contrast, the extended model was trained on the entire set of AP and AN variants, including those derived from sequencer-specific sequencing errors. Both training datasets were downsampled to 137, 500 samples each.

## Results

### Generation of a substantially extended dataset of actual positive and negative variants and training of a deep learning model

**Fig. 1:**
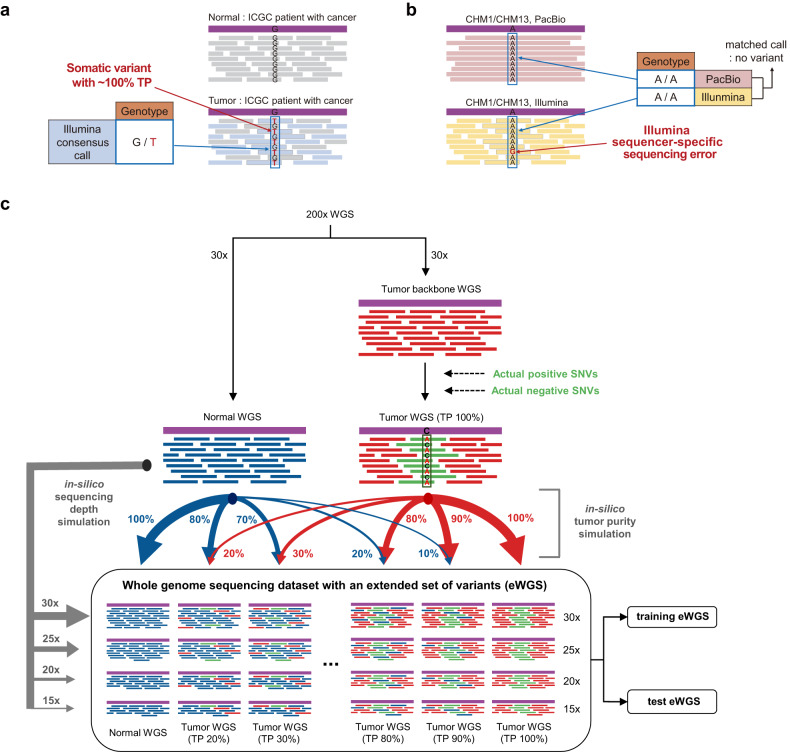
Generation of a substantially extended dataset of actual positive and negative variants. **a** The identification of actual positive single nucleotide variants (SNVs) from patients with cancer. Consensually-called somatic SNVs from ICGC tumor WGS data were utilized to identify the actual positive SNVs (see section “Materials and methods”). Sequencing reads derived from tumor cells are shown in light blue, while normal cells are shown in gray. **b** The identification of actual negative SNVs derived from Illumina sequencer-specific sequencing errors. The sequencing reads of CHM1/CHM13 from the Illumina and PacBio sequencers were compared to identify the actual negative SNVs (see section “Materials and methods”). The PacBio reads are shown in red, while the Illumina reads are shown in yellow. **c** Whole genome sequencing dataset with an extended set of variants (eWGS). By spiking a group of sequencing reads that encompassed actual positive and negative SNVs into a single 30x WGS backbone, the tumor WGS dataset was generated. For normal and tumor WGS data, various TP and SDs were simulated. The eWGS dataset was split into two independent datasets, the training and test eWGS data (see section “Materials and methods”).

To generate a high-quality dataset of actual positive (AP) variants, we obtained tumor WGS data for a large number of patients with cancer from the International Cancer Genome Consortium (ICGC). We then identified a subset of tumor WGS data with a tumor purity (TP) close to 100% by observing the overall distribution of variant allele frequencies (see section “Materials and methods”) and regarded the detected somatic variants in the WGS data as AP variants (Fig. 1a). As discussed earlier, WGS data generally include a large number of false positives originating from sequencer-specific sequencing errors. We collected Illumina sequencer-specific sequencing errors by comparing a pair of WGS data generated for a single sample both by Illumina and Pacific Bioscience (PacBio) sequencers and regarded these as actual negative (AN) variants (Fig. 1b). These AN variants showed a unique triplet nucleotide distribution compared to other variants (Supplementary Fig. 1). This finding suggests that a distinct and sequencer-specific process is likely responsible for the occurrence of these AN variants. By spiking a group of sequencing reads that encompassed the collected AP and AN variants into a single 30x WGS backbone, we generated simulated WGS data that included AP and AN variants as well as their paired normal WGS data. For each WGS pair, an in silico simulation, by serially mixing the tumor WGS data and their paired normal WGS data for various simulated relative fractions between two WGS datasets, was performed to generate simulated tumor WGS data that accurately reflected various TPs. Similarly, simulated tumor WGS data precisely accounting for various sequencing depths (SDs) were generated (Fig. 1c). Accordingly, these simulated WGS data included an extended set of AP variants that covers a wide range of TPs and SDs, as well as an extended set of AN variants derived from sequencer-specific sequencing errors. We named these simulated WGS data with an extended set of variants eWGS data. The eWGS data were expected to be enriched for AP and AN variants identified in tumor samples obtained from patients with actual cancer. By training a deep learning model on an extended set of variants from the eWGS dataset, AIVariant learned these complex and realistic features of actual tumor samples. AIVariant used two types of data matrices as inputs to the deep learning model. The first data matrix, termed an AIVariant image, included raw features of sequencing reads around a variant, representing a complete picture of alignments around the variant, but the number of reads that can be represented is limited by a certain fixed height, 100 in the current version of AIVariant, of the data matrix. On the other hand, the second data matrix, termed an AIVariant stat image, included summarized features of sequencing reads around a variant, designed to compensate for the limitation of the first data matrix by statistically summarizing features of all the aligned reads around the variant (Supplementary Fig. 2a). The deep learning model of AIVariant utilized residual network^26^ encoders for each of two data matrices and merged the encoded information with a fully connected network (Supplementary Fig. 2b).

### Accurate detection of somatic variants under low tumor purity and low sequencing depth

**Fig. 2:**
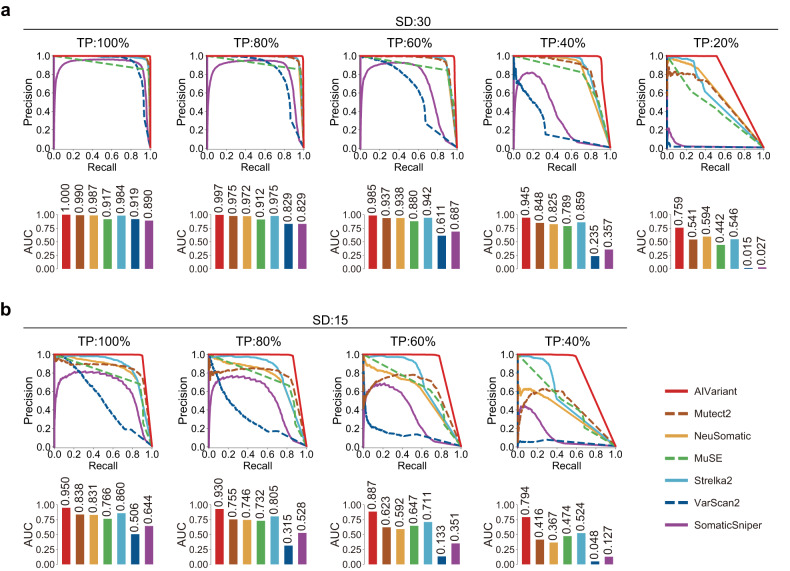
Somatic variant detection accuracy on test eWGS data. Evaluation of somatic variant detection accuracy on test eWGS data. The PR curve (top) and PR-AUC (bottom) are shown for each TP and SD case (see Supplementary Fig. 3 for cases not shown). **a** SD of 30 and TPs of 100, 80, 60, 40, and 20%; **b** SD of 15 and TPs of 100, 80, 60, and 40%.

To evaluate the somatic variant detection accuracy of AIVariant, we compared its accuracy with that of previously developed and widely used detectors, including Mutect2^15^, Strelka2^16^, MuSE^17^, VarScan2^18^, SomaticSniper^19^, and NeuSomatic^20,21^, on test eWGS data (see section “Materials and methods”). AIVariant outperformed the other detectors across all the examined TPs and SDs (Fig. 2a, b, and Supplementary Fig. 3a–d). Notably, AIVariant exhibited substantially high accuracy at low TPs and SDs. For instance, with 40% TP and an SD of 15, AIVariant achieved an area under the precision-recall curve (PR-AUC) of 0.794, whereas the other detectors achieved PR-AUC values of 0.048 to 0.524 (Fig. 2b). The difference in accuracy between AIVariant and the other detectors tended to increase as TP or SD decreased. With an SD of 30 and TPs of 100, 80, 60, 40, and 20%, the PR-AUCs of AIVariant were 0.010, 0.022, 0.043, 0.086, and 0.165 higher, respectively, than that of the second best-performing detector in each case. Similarly, with a 100% TP and SDs of 30, 25, 20, and 15, the PR-AUC difference between AIVariant and the second best-performing detector in each case was 0.010, 0.018, 0.044, and 0.090, respectively (Fig. 2a, b and Supplementary Fig. 3a–d). When evaluating test eWGS data for a specific cancer tissue type, AIVariant still demonstrated the highest PR-AUC across all the examined TPs and SDs, regardless of the tissue type. With a 100% TP and an SD of 30, AIVariant achieved PR-AUCs of ≥0.999 for each tissue type. With a 40% TP and an SD of 15, AIVariant achieved PR-AUCs of 0.804, 0.801, 0.763, 0.755, and 0.755 for pancreatic, liver, skin, ovarian, and pediatric brain cancers, respectively (Supplementary Fig. 3e, f).

### Validation of somatic variant detection accuracy on independent public datasets

**Fig. 3:**
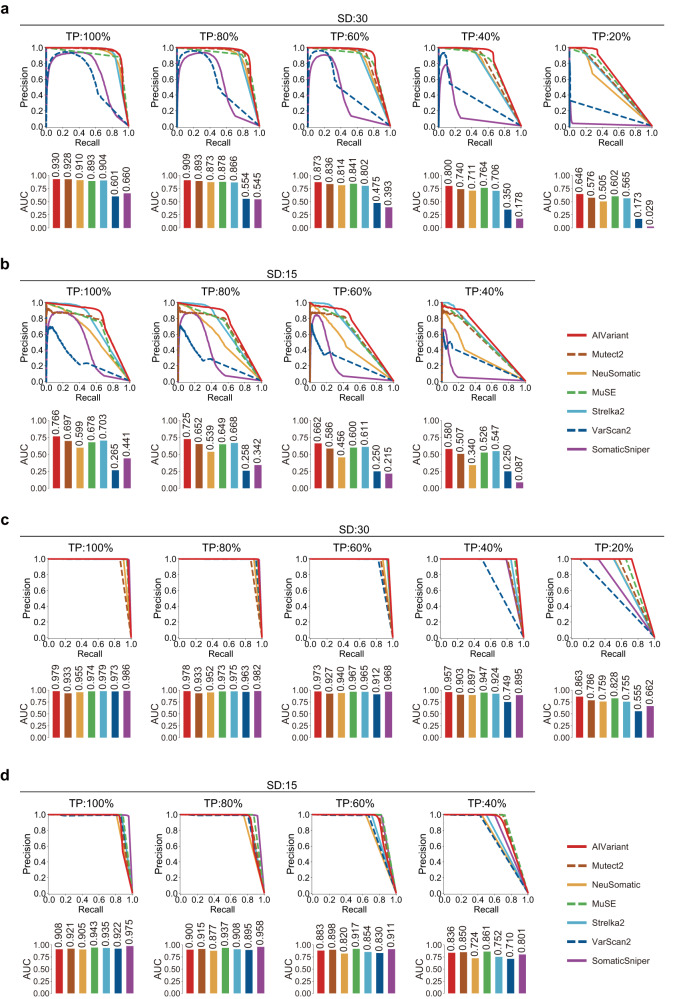
Validation of somatic variant detection accuracy on public benchmark datasets. Evaluation of somatic variant detection accuracy on the DREAM-Challenge and Platinum Genomes datasets (see Supplementary Fig. 4 for cases not shown). Accuracy for the DREAM-Challenge dataset. **a** SD of 30 and TPs of 100, 80, 60, 40, and 20%; **b** SD of 15 and TPs of 100, 80, 60, and 40%. Accuracy for the Platinum Genomes dataset. **c** SD of 30 and TPs of 100, 80, 60, 40, and 20%; **d** SD of 15 and TPs of 100, 80, 60, and 40%.

To demonstrate the generalizability of AIVariant, we evaluated its accuracy on two publicly available benchmark datasets, DREAM-Challenge^25^ and Platinum Genomes^22^. Both are widely used for the evaluation of somatic variant detection accuracy^16,20^ and are completely independent of AIVariant because they are not used in its training step. Again, AIVariant outperformed the other detectors across all the examined TPs and SDs on the DREAM-Challenge dataset evaluation (Fig. 3a, b and Supplementary Fig. 4a–d). For example, with a 100% TP and an SD of 30, AIVariant achieved a PR-AUC of 0.930, whereas the other detectors achieved PR-AUCs of 0.601–0.928 (Fig. 3a). With a 40% TP and an SD of 15, AIVariant achieved a PR-AUC of 0.580, whereas the other detectors achieved PR-AUCs ranging from 0.087 to 0.547 (Fig. 3b). AIVariant demonstrated the best accuracy for 17 out of 34 cases in the Platinum Genomes dataset evaluation. Although AIVariant was not always the top performer, the measured accuracy difference between AIVariant and the best-performing detector in each case was negligible (Fig. 3c, d and Supplementary Fig. 4e–h). Most detectors exhibited high accuracy on the Platinum Genomes dataset compared to the test eWGS and DREAM-Challenge datasets. This is perhaps due to inflated precision caused by the unrealistically high number of AP variants in the Platinum Genomes dataset (see section “Discussion”).

### The impact of the extended dataset of actual positive and negative variants on somatic variant detection

**Fig. 4:**
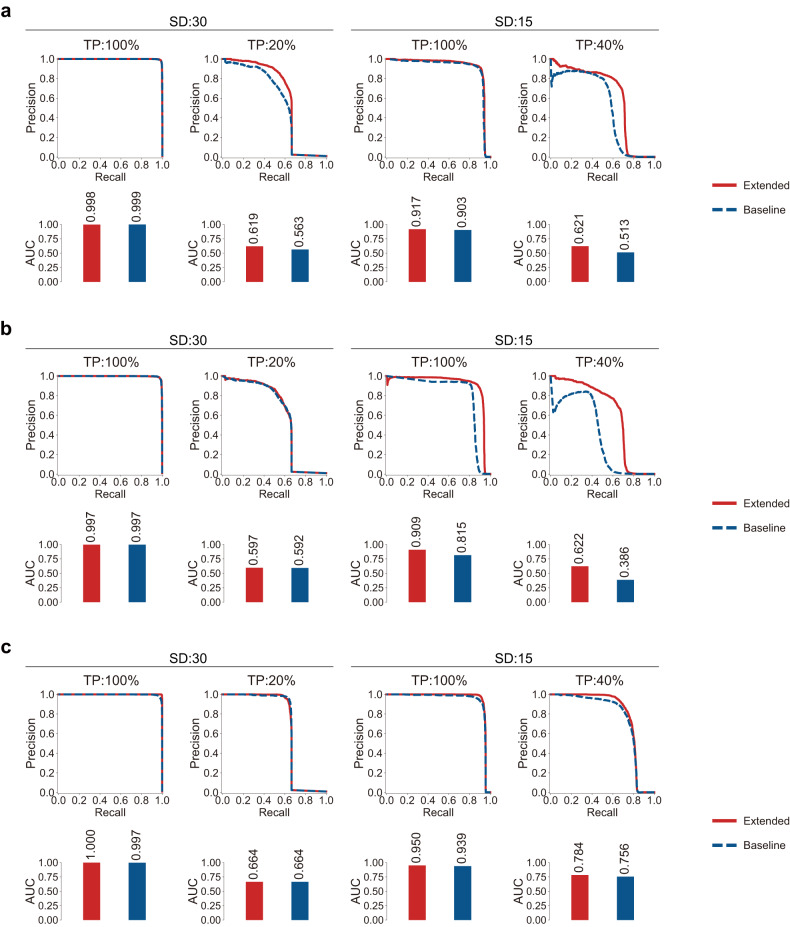
The impact of the extended set of variants on somatic variant detection. Accuracy comparison between the baseline model trained on datasets, where considerations for one of the three factors (various TPs, SDs, and sequencer-specific errors) are excluded, and the extended model trained on datasets, which takes all three factors into account. Various cases of SD and TP are compared. Precision-recall curves comparing the accuracies of the extended model and the baseline model **a** excluding various TPs, **b** excluding various SDs, and **c** excluding sequencer-specific sequencing errors.

We designed three analyses to investigate the impact of crucial factors that were systematically simulated in the extended set of variants: (1) various TPs, (2) various SDs, and (3) sequencer-specific sequencing errors. Each analysis compares the accuracy of two models: the extended model that incorporates all three factors and the baseline model that takes only two of the three factors into account (see section “Materials and methods”). Using PR-AUC as the accuracy metric, we compared two models on the test eWGS data in various cases: SD of 30 and 100% TP, SD of 30 and 20% TP, SD of 15 and 100% TP, and SD of 15 and 40% TP. In the first analysis, the extended model that took various TPs into account exhibited significantly better accuracies for low TPs than the baseline model, while the accuracies of the two models for high TPs showed marginal differences (Fig. 4a). This illustrates the effectiveness of the extended set, as the training dataset of the extended model had a significantly lower number of AP and AN variants from high TPs than the baseline model, which exclusively consisted of AP and AN variants from high TPs. The second analysis yielded similar results: the extended model, which considered various SDs, exhibited significantly better accuracies for low SDs than the baseline model, while the differences in accuracies for high SDs were marginal (Fig. 4b). The third analysis showed that including AN variants derived from sequencer-specific errors led to a detectable improvement in accuracy, as the extended model had better accuracies across all the evaluated cases (Fig. 4c). Taken together, these results explicitly illustrate that the extended set of variants, which encompasses a broad range of TPs, SDs, and sequencing errors specific to the sequencer, enhances the overall detection accuracy for somatic variants, particularly in tumor samples with lower TPs or SDs.

## Discussion

The observed higher detection accuracy of AIVariant emphasizes the significance of a more comprehensive training dataset that consists of a wide range of TPs, SDs, and sequencer-specific sequencing errors, reflected in the extended set of variants for AIVariant. In particular, the high accuracy of AIVariant for low SDs suggests a cost-effective solution for cancer diagnosis by allowing the use of lower SDs when generating WGS data without a sacrifice in detection accuracy, highlighting the potential clinical utility of our method. AIVariant incorporated Illumina sequencer-specific sequencing errors in comparison with PacBio WGS data, improving the detection accuracy across various TPs and SDs. This approach can be easily applied to include frequent sequencing errors from other sequencers such as PacBio, MGI, and Oxford Nanopore by using WGS data from these sequencing platforms. To the best of our knowledge, previous methods have not incorporated sequencer-specific sequencing errors in such a systematic and scalable manner. While AIVariant outperformed the other methods on most of the examined datasets, a few methods showed better accuracy, albeit marginal, on the Platinum Genomes dataset. This could be attributed to the presence of an unrealistically high number of AP variants in the Platinum Genomes dataset, ~1.2 million AP variants per sample in contrast to ~5000 AP variants per sample for the ICGC/TCGA Pan-Cancer Analysis of Whole Genome Consortium^24^, which may have allowed other methods to leniently predict as many variants as possible, thereby overly inflating precision. Methods that apply this kind of strategy would experience deteriorating precision on datasets with more realistic numbers of AP variants. Indeed, these methods exhibited lower detection precisions and overall accuracies when evaluated on our test eWGS data and the DREAM-Challenge dataset, which include more realistic numbers of AP variants than the Platinum Genomes dataset. Both the eWGS and DREAM-Challenge datasets have ~6000 AP variants per sample. In this study, our method focused on single nucleotide variations (SNVs), since SNVs are the most frequently discovered and thus most responsible type of somatic variants in cancer genomes^27,28^. However, other types of somatic variants, such as small insertions/deletions and structural variations, may additionally hold crucial information for the cancer genome landscape. Because our current deep-learning approach that constructs an extended set of variants can be easily applied to other variant types, we are planning to expand our approach to other types of variants to make AIVariant more comprehensive and accurate than the current version. In conclusion, AIVariant demonstrates state-of-the-art accuracy for somatic variant detection and therefore is expected to be useful for cancer research, diagnosis, and therapeutics.

## Supplementary information

Supplementary information for AIVariant: a deep learning-based somatic variant detector for highly contaminated tumor samples

## Data Availability

The source code and data are available on GitHub (https://github.com/Genome4me/AIVariant).

## Code availability

The source code and data are available on GitHub (https://github.com/Genome4me/AIVariant).
