# Author Correction: AIVariant: a deep learning-based somatic variant detector for highly contaminated tumor samples

**DOI:** 10.1038/s12276-025-01405-4

**Published:** 2025-01-28

**Authors:** Hyeonseong Jeon, Junhak Ahn, Byunggook Na, Soona Hong, Lee Sael, Sun Kim, Sungroh Yoon, Daehyun Baek

**Affiliations:** 1 Interdisciplinary Program in Bioinformatics, Seoul National University, Seoul, Republic of Korea; 2 Genome4me Inc., Seoul, Republic of Korea; 3 School of Biological Sciences, Seoul National University, Seoul, Republic of Korea; 4 Department of Electrical and Computer Engineering, Seoul National University, Seoul, Republic of Korea; 5 AIGENDRUG Co., Ltd., Seoul, Republic of Korea; 6 Department of Software and Computer Engineering, Ajou University, Suwon, Republic of Korea; 7 Department of Computer Science and Engineering, Seoul National University, Seoul, Republic of Korea; 8 Interdisciplinary Program in Artificial Intelligence, Seoul National University, Seoul, Republic of Korea

**Keywords:** Machine learning, Cancer, Data processing

Correction to: *Experimental & Molecular Medicine* (2023) 55: 1734–1742 10.1038/s12276-023-01049-2; Article published online 01 August 2023

In this Article, the funding from ‘the Technology development Program, funded by the Ministry of SMEs and Startups, Republic of Korea (RS-2023-00258711)’ was omitted.

The correct statement of this Article should have read as below.

“We express our gratitude to Sangho Park from Genome4me Inc. for valuable advice and fruitful discussions regarding software development and optimization for AIVariant. This study was supported by the National Research Foundation of Korea (NRF), funded by the Ministry of Science and ICT, Republic of Korea (NRF-2014M3C9A3063541, NRF-2019M3E5D3073104, NRF-2020R1A2C3007032, NRF-2020R1A5A1018081, and NRF-2022M3A9I2082294), by the Korea Health Industry Development Institute (KHIDI), funded by the Ministry of Health and Welfare, Republic of Korea (HI15C3224), by the Technology development Program, funded by the Ministry of SMEs and Startups, Republic of Korea (RS-2023-00258711), and by KREONET (Korea Research Environment Open NETwork), managed and operated by KISTI (Korea Institute of Science and Technology Information).”

The original Article has been corrected.

