# Supplementary material for: AIVariant: a deep learning-based somatic variant detector for highly contaminated tumor samples
Source: Exp Mol Med. 2023 Aug 1;55(8):1734–42. doi: 10.1038/s12276-023-01049-2 (PMC10474289; doi:10.1038/s12276-023-01049-2)
Supplement: Supplementary file 1 — Supplementary information for AIVariant: a deep learning-based somatic variant detector for highly contaminated tumor samples [file 12276_2023_1049_MOESM1_ESM.pdf]

AIVariant: a deep learning-based somatic variant detector for highly contaminated tumor samples

Hyeonseong Jeon[1,2,*], Junhak Ahn[2,3,*], Byunggook Na[4], Soona Hong[5], Lee Sael[6], Sun Kim[7], Sungroh Yoon[4,8], and Daehyun Baek[1,2,3,8,9]

[1]Interdisciplinary Program in Bioinformatics, Seoul National University, Seoul 08826, Republic of Korea.

[2]Genome4me Inc., Seoul 08826, Republic of Korea.

[3]School of Biological Sciences, Seoul National University, Seoul 08826, Republic of Korea.

[4]Department of Electrical and Computer Engineering, Seoul National University, Seoul 08826, Republic of Korea.

[5]AIGENDRUG Co., Ltd., Seoul 08826, Republic of Korea.

[6]Department of Software and Computer Engineering, Ajou University, 16499, Suwon, Republic of Korea.

[7]Department of Computer Science and Engineering, Seoul National University, Seoul 08826, Republic of Korea.

[8]Interdisciplinary Program in Artificial Intelligence, Seoul National University, Seoul 08826, Republic of Korea.

[9]Correspondence should be addressed to D.B. (baek@snu.ac.kr).

[*]These authors contributed equally to this work.

This PDF file includes:

Supplementary Materials and Methods

Supplementary Table 1

Supplementary Figure legends

Supplementary Figures 1-5

References

Supplementary Materials and Methods

AIVariant preprocessing

The inputs for preprocessing were aligned sequencing read BAM files of paired tumor and normal samples. As the initial preprocessing step, the 'Picard MarkDuplicates (v2.18.1)' was applied to the input BAM files. Low-quality reads, including reads that were unmapped, PCR or optically duplicated, had secondary alignments, and had a mapping quality of 0 were then filtered out. The final preprocessing step was the base quality normalization, in which the base quality distribution of the BAM file was made to follow the reference base quality distribution (Supplementary Fig. 5a). To remove potential base quality bias derived from specific research group (Supplementary Fig. 5b and c), AIVariant uses normalized base qualities in AIVariant somatic variant detection. This was achieved by randomly scanning reads, totaling ten million bases, in the BAM file to obtain the base quality distribution of the sequencing data of the target sample. The base quality of each read in the BAM file was then converted to exactly match the reference base quality distribution. To do so, the reference base quality distribution and the target sample base quality distribution were first sorted by base quality. The highest entry in the target sample's base quality distribution was then assigned the value of the highest base quality in the reference distribution, followed by the next highest base quality, until the target sample distribution was aligned with the reference distribution. The resultant correspondence was used to convert the base quality of all reads in the target sample. The reference base quality distribution utilized for base quality normalization was derived by averaging the base quality distribution of 81 ICGC tumor BAM files (Supplementary Fig. 5c), and sorted by base quality.

AIVariant candidate search

A candidate position should satisfy the following:

$$\begin{cases} B_{tumor}(alt) - B_{normal}(alt) \geq 35 * depth_{tumor} * \dfrac{2}{30} \\ B_{normal}(alt) < 30 * depth_{normal} * \dfrac{1}{30} \\ depth_{tumor}(position) \geq \dfrac{depth_{tumor}}{3} \ \ and \ \ depth_{normal}(position) \geq \dfrac{depth_{normal}}{3} \end{cases}$$

Where, $B_{tumor/normal}(x)$ is a sum of base qualities for base x in tumor/normal BAM, ref is defined as the base of reference sequence at a given genomic position, alt is defined as, $\text{argmax}_{x \in \{A,C,G,T | x \neq ref\}} B_{tumor}(x) - B_{normal}(x)$ and called "max alternate allele", $depth_{tumor/normal}$ is a sequencing depth of tumor/normal BAM, $depth_{tumor/normal}(position)$ is a number of aligned reads that covers a given position in tumor/normal BAM. In addition to this, a candidate position should satisfy two more criteria:

$\begin{cases} LODn \leq 0.05 \\ depth_{normal}(position) \geq \frac{depth_{normal}}{3} \end{cases}$, and see **AIVariant inputs** for the definition of LODn.

For train eWGS, we applied a slightly different condition of $depth_{normal}(position) \geq 10$, since an insufficient number of reads can lead to unstable training.

AIVariant inputs

AIVariant inputs consisted of two types of three-dimensional matrices, AIVariant images and AIVariant stat images, as well as four log odds (LODs) values.

The three-dimensional matrices, hereafter images, were constructed from the read collections. AIVariant has four types of read collections: the raw tumor read, raw normal read, processed tumor read, and processed normal read collections. For a given candidate position, we collected

reads aligned to its 50 bases flanking region. Reads collected from the tumor sequencing data were called the raw tumor read collection, while reads from normal sequencing data were called the raw normal read collection. The processed tumor/normal read collection was a subcollection of tumor/raw normal read collections that held high-quality reads that satisfied specific criteria. The processed normal read collection consisted of reads in the raw normal read collection that satisfied the following criteria: 1) a read did not have a deletion at the candidate position; 2) a read was aligned to the candidate position with a base quality ≥ 5. The processed tumor read collection consisted of reads in the raw tumor read collection that satisfied the above criteria and also satisfied the following: 1) a read had mapping quality > 0; 2) a ratio of soft-clipped length over read length was < 0.3; 3) the sum of base qualities for mismatches was < 100.

An AIVariant image was constructed from each read collection; therefore, we constructed four AIVariant images for a given candidate position. Each collection was sorted according to the tuple key consisting of Boolean allele, mapping quality, and base quality at the candidate position, in this order, where the Boolean allele was 1 if the aligned read base at the candidate position was the same as "max alternate allele" and 0 otherwise. We used only the first 100 reads from each sorted collection to construct an AIVariant image, i.e., a three-dimensional matrix with dimensions of 100 × 101 × 16. One hundred rows represented the first 100 reads from a sorted collection, and if a collection had less than 100 reads, then the remaining rows were padded with zeroes. A total of 101 columns represented alignment columns, which were the extended reference genomic positions that included insertion information. Each alignment column could represent the reference position or insertion in alignment, and we extended the reference genomic position by taking insertions in at least one of the aligned reads in four collections when constructing alignment column insertions with lengths > 1, they were left aligned for a consistent extension. From this extended genomic position or alignment columns,

we took a window of length 101 centered at the candidate position, which was represented by 101 columns in the AIVariant image. Sixteen channels represented features relevant to somatic variants. The first four channels held one-hot encoded vectors for the reference A/C/G/T bases, and the next four channels held one-hot encoded vectors for the read A/C/G/T bases. The remaining channels represented mapping quality, original base quality, normalized base quality, read strand, soft-clip, distance from read ends, and epigenetic features. The mapping quality was min-max normalized to the [-1, 1] range by taking 0 as the minimum and 60 as the maximum. The base quality was min-max normalized to the [-1, 1] range by taking 0 as the minimum and 41 as the maximum. The distance from the read ends was the minimum distance of the given column position from the 5' read end and 3' read end, and the minimum was divided by half of the read length for normalization. Epigenetic features were obtained from the peak calling score and the p-value of H3K9me3 modification peak calling data generated by the Roadmap Epigenomics project[1]. We used H3K9me3 modification because it is reportedly one of the most relevant epigenetic features[2].

An AIVariant stat image was constructed from each processed tumor/normal read collection. For a given candidate position, we generated two AIVariant stat images of dimension 5 × 101 × 9, and an AIVariant stat image was constructed from the whole reads of a read collection, different from AIVariant images, which were constructed from only 100 reads in a read collection. Five rows represented A/C/G/T/- bases and "-" was a gap base. As in the AIVariant images, 101 columns represented 101 alignment columns. Nine channels represented statistical values relevant to the somatic variants. The first channel held reference base information; for instance, if an alignment column represented the actual reference position, i.e., not a gap position, then a row representing this reference base took the value of 1 and the other rows in this column and the channel took a value of 0. If an alignment column represented a gap base, there was no reference base information for this column so only the row representing "-", a gap

base, took a value of 1 and other rows held a value of 0. The second channel was for the read base frequency; we counted the number of occurrences of A/C/G/T/- bases at this column position in aligned reads, where the gap base represents deletion in aligned reads, and divided each count by the number of reads that covered this column position. The remaining channels represented the averages of mapping quality, original base quality, normalized base quality, read strand, and distance to read ends, as well as epigenetic features. Averages of base quality and mapping quality were taken over reads covering a column position, and averages of other features were taken over each A/C/G/T/- base.

The LOD values are the likelihood values introduced by Mutect[3]. In AIVariant, we calculated a simplified version of Mutect LOD values to capture features relevant to somatic variants. We calculated four LOD values, namely LODt, LODn, unnormalized LODt, and unnormalized LODn, from the processed tumor/normal read collections. Similar to AIVariant stat images, LOD values were calculated using the whole reads in a collection. LODt and LODn were calculated using normalized base qualities, and unnormalized LODt and unnormalized LODn were calculated using the original base qualities. For a given candidate position, $LOD(v) \coloneqq \Sigma_{b \in B} log_{10} L(b; v)$, B is a set of read bases at the candidate position, L is a likelihood function defined as $L(b; v) \coloneqq \begin{cases} v * \frac{\epsilon}{3} + (1 - v) * (1 - \epsilon),\ if\ \ b = ref \\ v * \epsilon + (1 - v) * \frac{\epsilon}{3},\ if\ \ b = alt \\ \frac{\epsilon}{3},\ otherwise \end{cases}$, where $\epsilon \coloneqq 10^{-qual(b)/10}$, where $qual(b)$ is a base quality for b, so ϵ is an error probability for a base b, ref is a reference base at the candidate position, and alt is a max alternate allele at the candidate position. Finally, $LODt \coloneqq LOD(\ fraq_{alt}) - LOD(0)$, where $frac_{alt}$ is the frequency of max alternate allele and $LODn \coloneqq LOD(0) - LOD(0.5)$. $LODt$ is a simple measure of the log likelihood of variants supported by tumor sequencing data, and $LODn$ is a simple measure of the log likelihood of non-variants supported by normal data. LODt values

were min-max normalized with a maximum of 100, and LODn values were min-max normalized with a maximum of 50.

AIVariant architecture

The AIVariant deep neural network model consisted of two types of encoders and a merge layer followed by fully connected layers. The model input consisted of four AIVariant images, raw tumor, processed tumor, raw normal, and processed normal images; two AIVariant stat images, processed tumor and processed normal stat images; and four LOD values, LODt, LODn, unnormalized LODt, and unnormalized LODn. Both AIVariant encoders had the same residual network architecture, except for the input shape. One type of encoder encoded each AIVariant image into a vector of size 16, and another encoded each AIVariant stat image into a vector of size 16. Each LOD pair, LODt-LODn pair, and unnormalized LODt-unnormalized LODn pair were combined and extended to a vector of size 16 through a fully connected layer. The resulting eight vectors of size 16 were merged to obtain a vector of size 128 that went through fully connected layers. Finally, the merged vector was activated with the softmax function to output the posterior probability of the somatic variant at a given position. Overall, the AIVariant is a residual network model with multiple three-dimensional matrix inputs with approximately three million trainable parameters.

AIVariant training

For AIVariant training, we generated images from the train eWGS. As described above, four AIVariant images, two AIVariant stat images, and four LOD values were generated for each candidate position (see **AIVariant inputs**). The candidate positions for training were identified using the candidate search step, as previously described (see **AIVariant candidate search**).

To ensure stability in the AIVariant training, we balanced the number of actual positive(AP) variants and actual negative(AN) variants from the ten different types (9 cancer types from ICGC and one from CHM1/CHM13) represented in the train eWGS for each SD and TP. Balancing was performed for stable AIVariant training; however, we did not exactly match the numbers during balancing because this resulted in only 38 000 remaining training data, which was too few to train the model well. Therefore, we balanced the training data as much as possible while retaining > 100 000 training data. The final balanced training dataset contained 138 000 data points (Supplementary Table 1). We used 20% of the training data for validation with a batch size of 64 and Adam optimizer with an initial learning rate of 3*1e-3. The epoch was 100, and we only kept the weights of the best-performing model for the validation data.

Extrapolation of PR curve

A somatic variant detection method outputs scores only for the variants it deemed highly probable somatic variants. These scored variants only include a subset of AP somatic variants, and thus the maximum recall value a method can reach is limited. Hence, the PR curve, based on the output scores of a method, suddenly stops at this recall limit, middle of the precision-recall plane. Moreover, each somatic variant detection method has a different recall limit value due to different definitions of "highly probable" somatic variants. To overcome this complication when comparing PR-AUC, we extrapolated each PR curve by drawing a linear line between the recall limit point (the recall limit value and the corresponding precision value) and the bottom-right corner of the precision-recall plane (recall 1.0 and precision 0.0).

The extrapolation is achieved by adding enough dummy true positives and false positives with the specific score, which is less than the minimum score provided by each method. The number of dummy true positives and false positives was determined as follows:

the number of AP variants – the number of true positives = the number of dummy true positives.

99 × the number of AP variants – the number of false positives = the number of dummy false positives.

Supplementary Table 1. The number of AP variants and AN variants in train eWGS

Tumor purity	Sequencing depth	AN variants derived from Illumina sequencer-specific errors	AN variants originating from the random shuffling of reads	AP variants from somatic variants of patients with cancer	AP variants simulated by Illumina sequencer-specific errors-free germline variants
100%	30	1000	1000	1800	200
	25	1000	1000	1800	200
	20	1000	1000	1800	200
	15	1000	1000	1800	200
90%	30	1000	1000	1800	200
	25	1000	1000	1800	200
	20	1000	1000	1800	200
	15	1000	1000	1800	200
80%	30	1000	1000	1800	200
	20	1000	1000	1800	200
	25	1000	1000	1800	200
	15	1000	1000	1800	200
70%	30	1000	1000	1800	200
	20	1000	1000	1800	200
	25	1000	1000	1800	200
	15	872	1000	1800	200
60%	30	1000	1000	1800	200
	20	1000	1000	1800	200
	25	1000	1000	1800	200
	15	707	1000	1800	200
50%	30	1000	1000	1800	200
	25	1000	1000	1800	200
	20	938	1000	1800	200
	15	579	1000	1800	200
40%	30	1000	1000	1800	200
	25	1000	1000	1800	200
	20	724	1000	1800	200
	15	450	1000	1800	200
30%	30	614	1000	1800	200
	25	737	1000	1800	200
	20	542	1000	1800	200
	15	347	1000	1800	200
20%	30	442	1000	1800	200
	25	513	1000	1800	200
	20	389	1000	1800	200
	15	278	1000	1800	200

Supplementary Fig. 1. Trinucleotide distribution of actual positive and actual negative variants

Trinucleotide distribution of actual positive (AP) and actual negative (AN) variants used to train the AIVariant deep neural network (Supplementary Table 1). A trinucleotide was determined as nucleotides from one nucleotide upstream to one nucleotide downstream from an AP variant or AN variant position. The distribution is displayed as in a previous study[4].

Supplementary Fig. 2. AIVariant workflow

(a) Somatic variant detection process of AIVariant and images used for somatic variant calling. During the four key steps of the detection process (preprocessing, candidate search, image generation, and somatic variant calling steps), sequencing reads in tumor/normal sample BAM files are preprocessed and somatic variant candidates are identified. Images are then generated for the identified candidates and somatic variants are called based on the generated images.

(b) AIVariant inputs and architecture. The inputs consist of AIVariant images, AIVariant stat images, and LOD values. AIVariant image represents the complete picture of alignments around variant with raw features of sequencing reads, but the number of reads it can include is limited by the size of the image. AIVariant stat image summarized features of sequencing reads around a variant and compensate for the limitation of the AIVariant image by statistically summarizing features of all the reads. LOD values are log likelihoods of a variant and help stable training. Each image input is encoded by a residual network and LOD values are encoded by fully connected networks. Encoded vectors are merged and go through another fully connected network. The output is activated with softmax and this value represents the posterior probability for SNV.

Supplementary Fig. 3. Somatic variant detection accuracy on test eWGS

Evaluation of somatic variant detection accuracy on test eWGS for cases not shown in Fig. 2. Accuracy for (a) SD of 30 and TPs of 90, 70, 50, and 30%; (b) SD of 15 and TPs of 90, 70, and 50%; (c) SD of 25 and TPs of 90, 80, 70, 60, 50, 40, 30, and 20%; (d) SD of 20 and TPs of 90, 80, 70, 60, 50, 40, 30, and 20%. Accuracy for each of the specific eight cancer tissue types with (e) SD of 30 and 100% TP; (f) SD of 15 and 40% TP.

Supplementary Fig. 4. Validation of somatic variant detection accuracy on public benchmark datasets

Evaluation of somatic variant detection accuracy on the DREAM-Challenge and the Platinum Genomes datasets for cases not shown in Fig. 3. Accuracy for DREAM-Challenge dataset with (a) SD of 30 and TPs of 90, 70, 50, and 30%; (b) SD of 15 and TPs of 90, 70, and 50%; (c) SD of 25 and TPs of 90, 80, 70, 60, 50, 40, 30, and 20%; (d) SD of 20 and TPs of 90, 80, 70, 60, 50, 40, 30, and 20%; and for the Platinum Genomes dataset with (e) SD of 30 and TPs of 90, 70, 50, and 30%; (f) SD of 15 and TPs of 90, 70, and 50%; (g) SD of 25 and TPs of 90, 80, 70, 60, 50, 40, 30, and 20%; (h) SD of 20 and TPs of 90, 80, 70, 60, 50, 40, 30, and 20%.

Supplementary Fig. 5. AIVariant base quality normalization

(a) The reference base quality distribution utilized in the AIVariant base quality normalization.

(b) Base quality distributions of several WGS datasets used in this study, including CHM1-CHM13[5] (yellow), NA12877 200x (green)[6], tumor/normal of ICGC-TCGA DREAM Somatic Variant Calling Challenge Synthetic Data3[7] (blue), and, NA12877/NA12878 50x[6] (purple).

(c) Base quality distributions of 81 cancer tissue samples from nine ICGC projects (PBCA-DE, PAEN-AU, MALY-DE, LIRI-JP, OV-AU, MELA-AU, PACA-CA, PRAD-UK, and THCA-US)[8]. The donor, specimen, and sample IDs of each ICGC sample are shown in the upper left corner of the distribution panels.

Supplementary Fig. 1

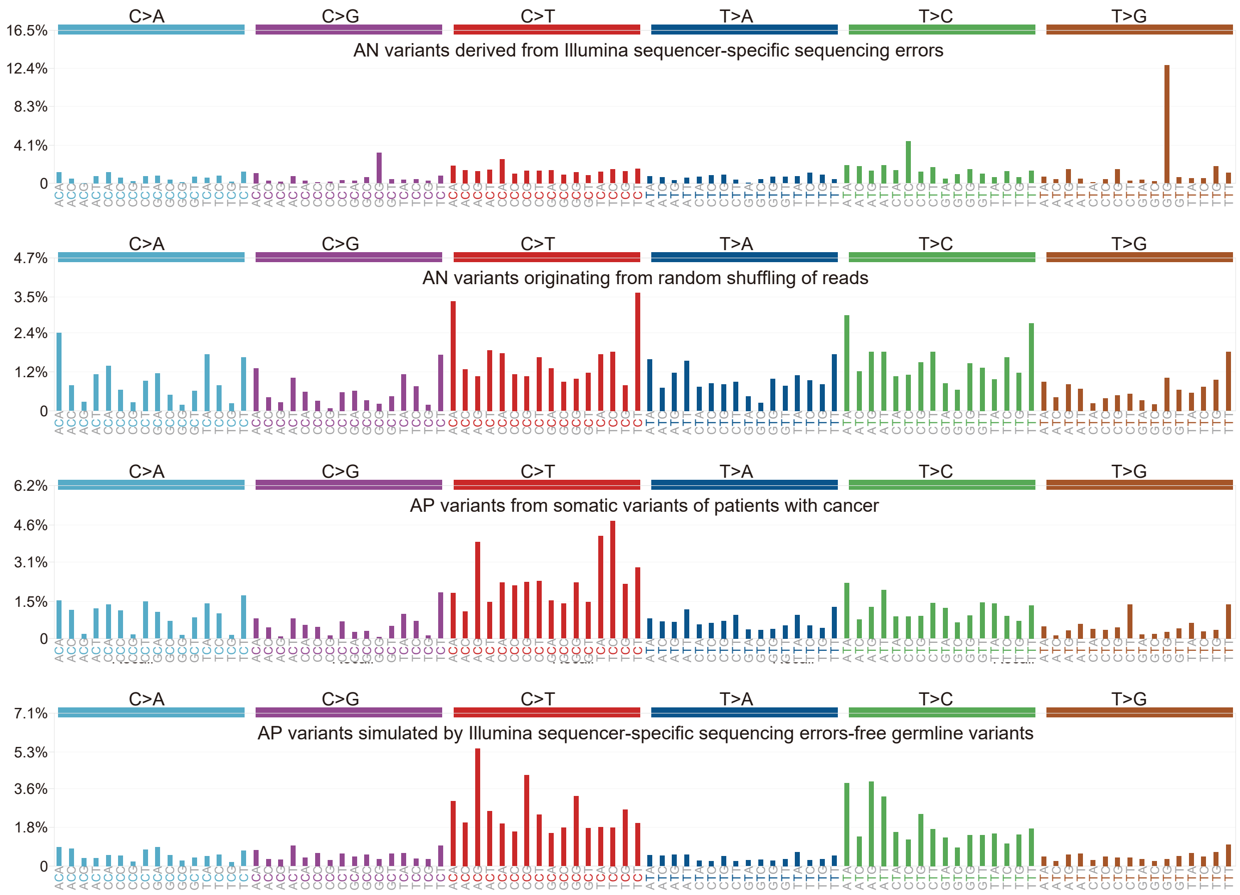

Supplementary Fig. 2

a

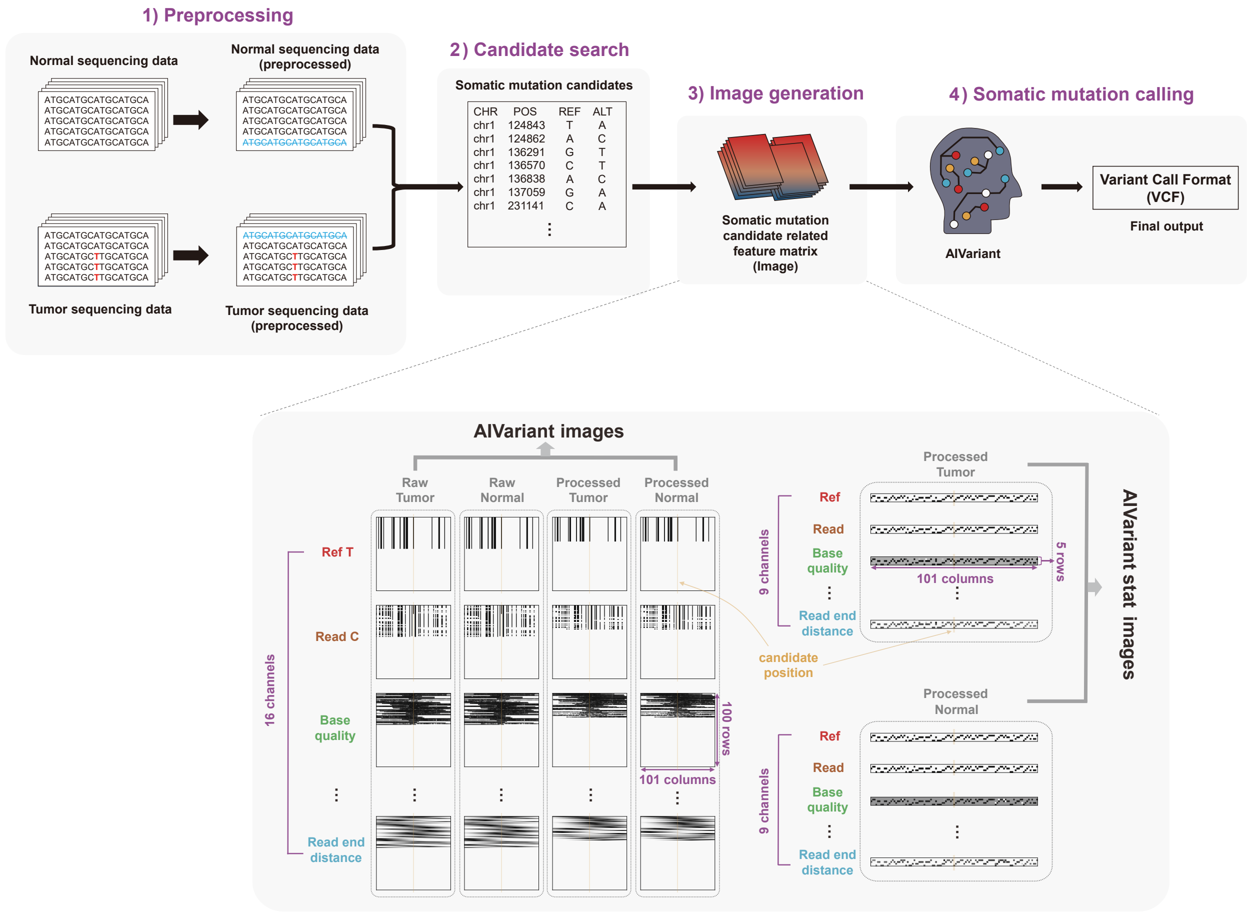

b

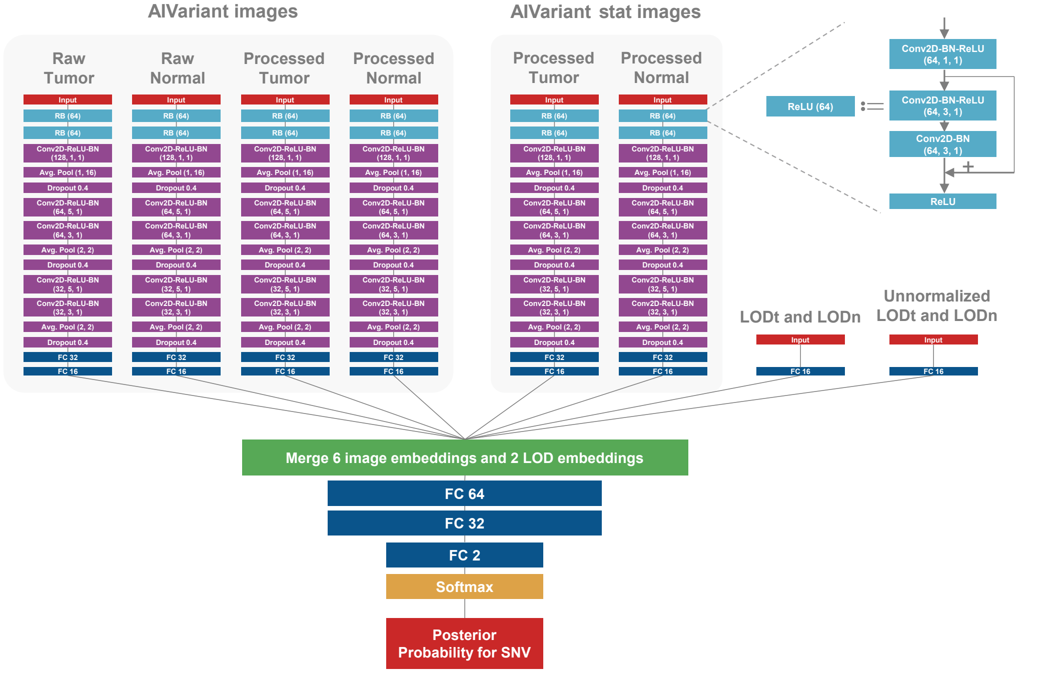

Supplementary Fig. 3

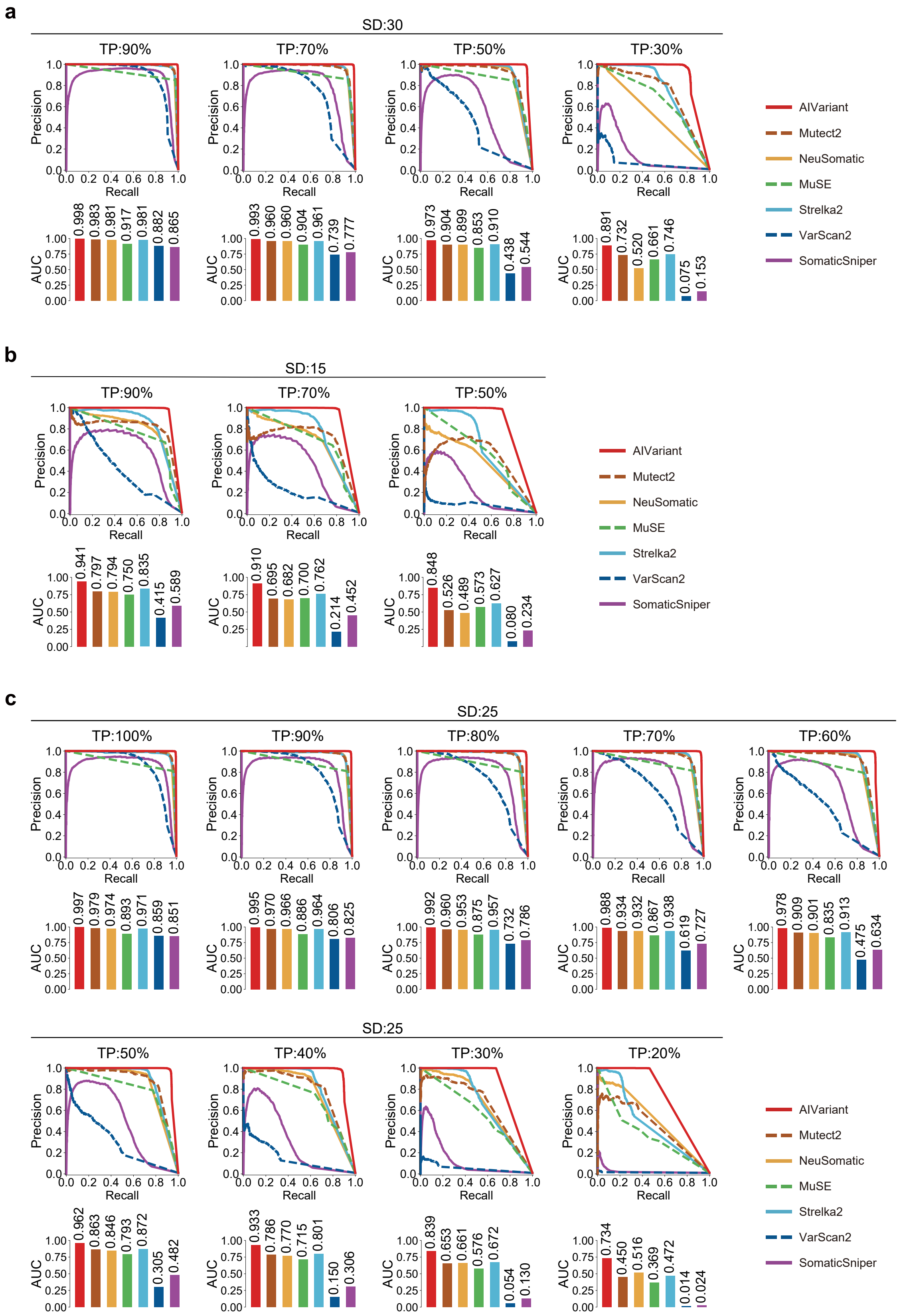

d

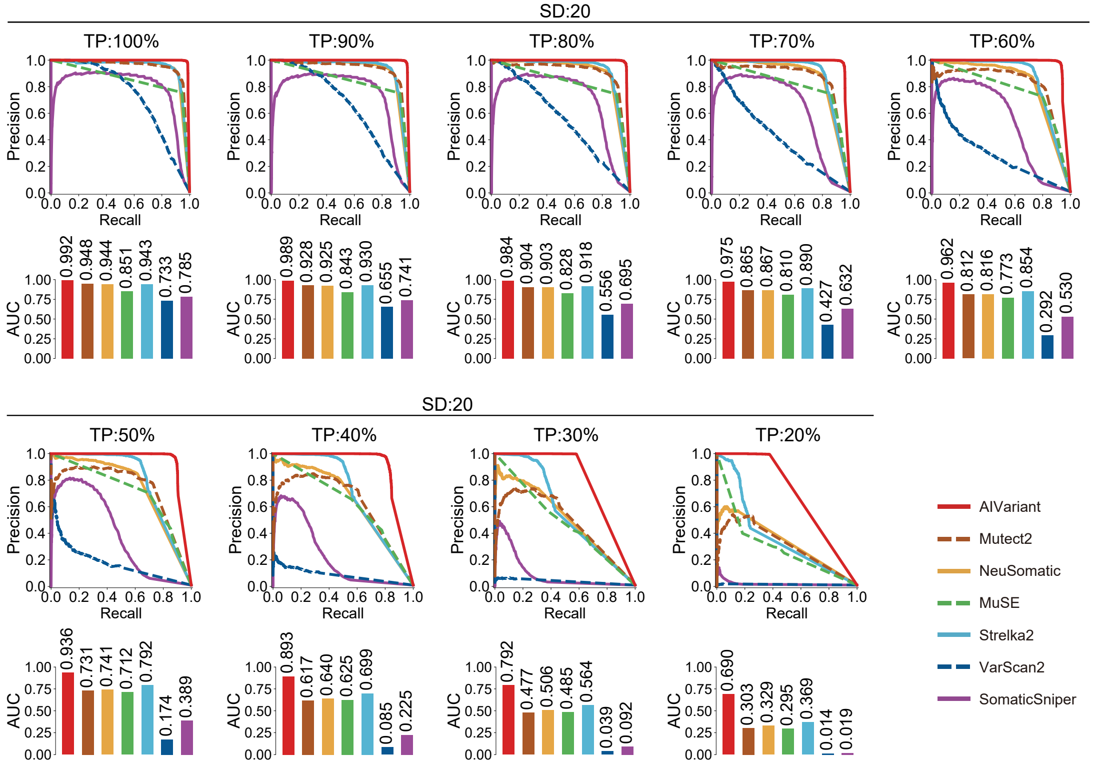
SD:20
TP:100%
TP:90%
TP:80%
TP:70%
TP:60%
Precision
Recall
AUC
0.992 0.948 0.944 0.851 0.943 0.733 0.785
0.989 0.928 0.925 0.843 0.930 0.655 0.741
0.984 0.904 0.903 0.828 0.918 0.556 0.695
0.975 0.865 0.867 0.810 0.890 0.427 0.632
0.962 0.812 0.816 0.773 0.854 0.292 0.530
SD:20
TP:50%
TP:40%
TP:30%
TP:20%
0.936 0.731 0.741 0.712 0.792 0.174 0.389
0.893 0.617 0.640 0.625 0.699 0.085 0.225
0.792 0.477 0.506 0.485 0.564 0.039 0.092
0.690 0.303 0.329 0.295 0.369 0.014 0.019
AIVariant
Mutect2
NeuSomatic
MuSE
Strelka2
VarScan2
SomaticSniper

e

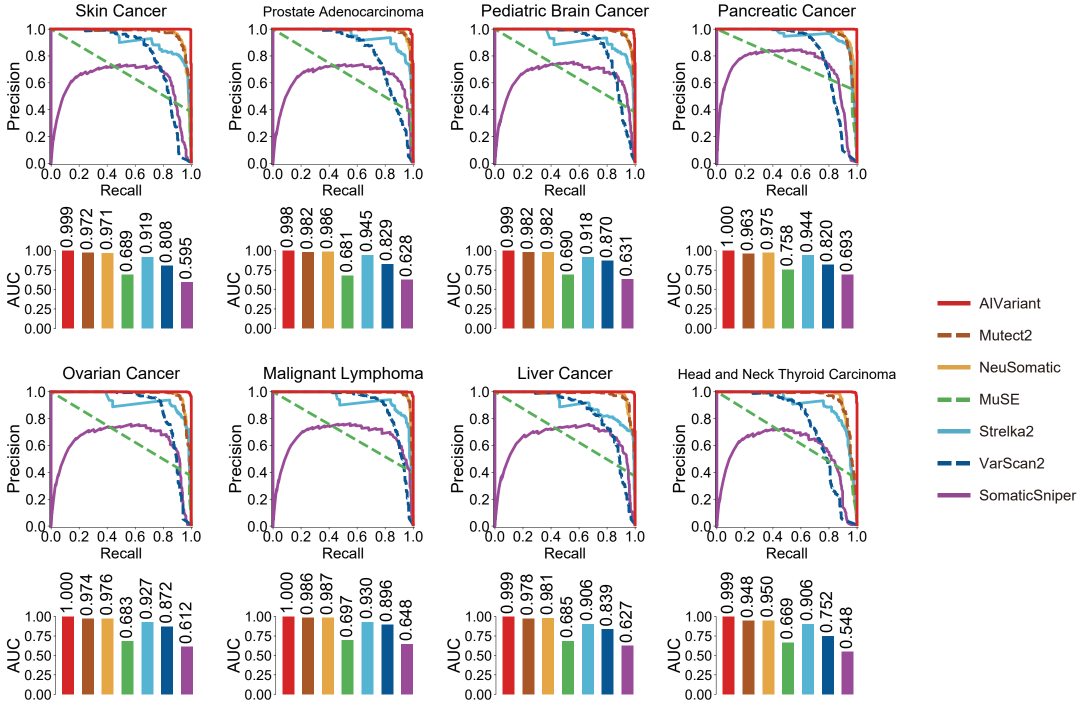
Skin Cancer
Prostate Adenocarcinoma
Pediatric Brain Cancer
Pancreatic Cancer
Precision
Recall
AUC
0.999 0.972 0.971 0.689 0.919 0.808 0.595
0.998 0.982 0.986 0.681 0.945 0.829 0.628
0.999 0.982 0.982 0.690 0.918 0.870 0.631
1.000 0.963 0.975 0.758 0.944 0.820 0.693
Ovarian Cancer
Malignant Lymphoma
Liver Cancer
Head and Neck Thyroid Carcinoma
1.000 0.974 0.976 0.683 0.927 0.872 0.612
1.000 0.986 0.987 0.697 0.930 0.896 0.648
0.999 0.978 0.981 0.685 0.906 0.839 0.627
0.999 0.948 0.950 0.669 0.906 0.752 0.548
AIVariant
Mutect2
NeuSomatic
MuSE
Strelka2
VarScan2
SomaticSniper

f

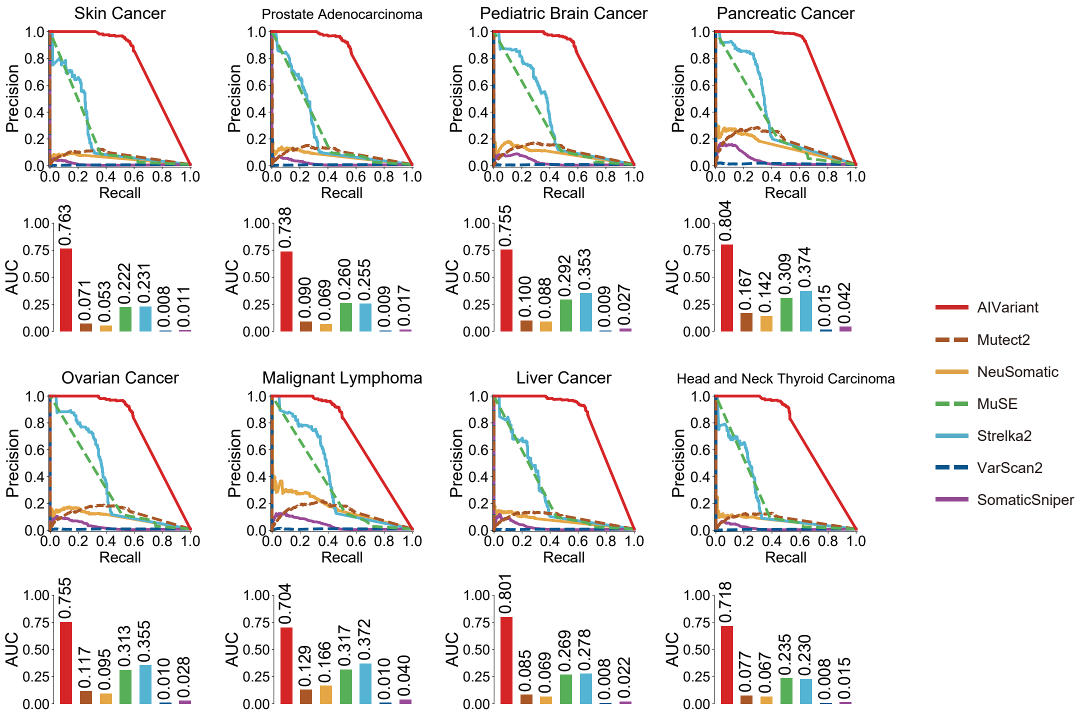
Skin Cancer
Prostate Adenocarcinoma
Pediatric Brain Cancer
Pancreatic Cancer
Ovarian Cancer
Malignant Lymphoma
Liver Cancer
Head and Neck Thyroid Carcinoma
Precision
Recall
AUC
0.763
0.071
0.053
0.222
0.231
0.008
0.011
0.738
0.090
0.069
0.260
0.255
0.009
0.017
0.755
0.100
0.088
0.292
0.353
0.009
0.027
0.804
0.167
0.142
0.309
0.374
0.015
0.042
0.755
0.117
0.095
0.313
0.355
0.010
0.028
0.704
0.129
0.166
0.317
0.372
0.010
0.040
0.801
0.085
0.069
0.269
0.278
0.008
0.022
0.718
0.077
0.067
0.235
0.230
0.008
0.015
AIVariant
Mutect2
NeuSomatic
MuSE
Strelka2
VarScan2
SomaticSniper

Supplementary Fig. 4

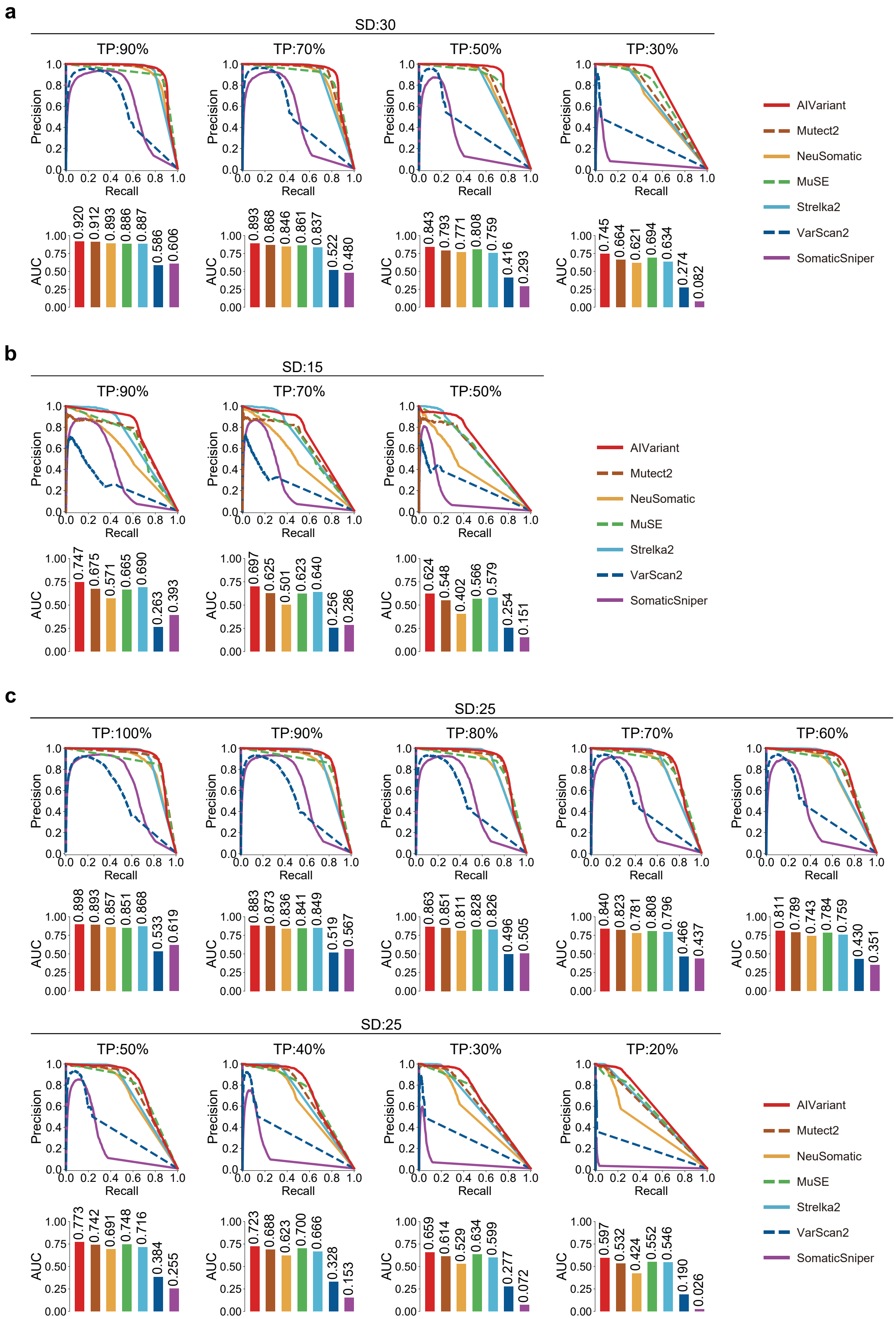

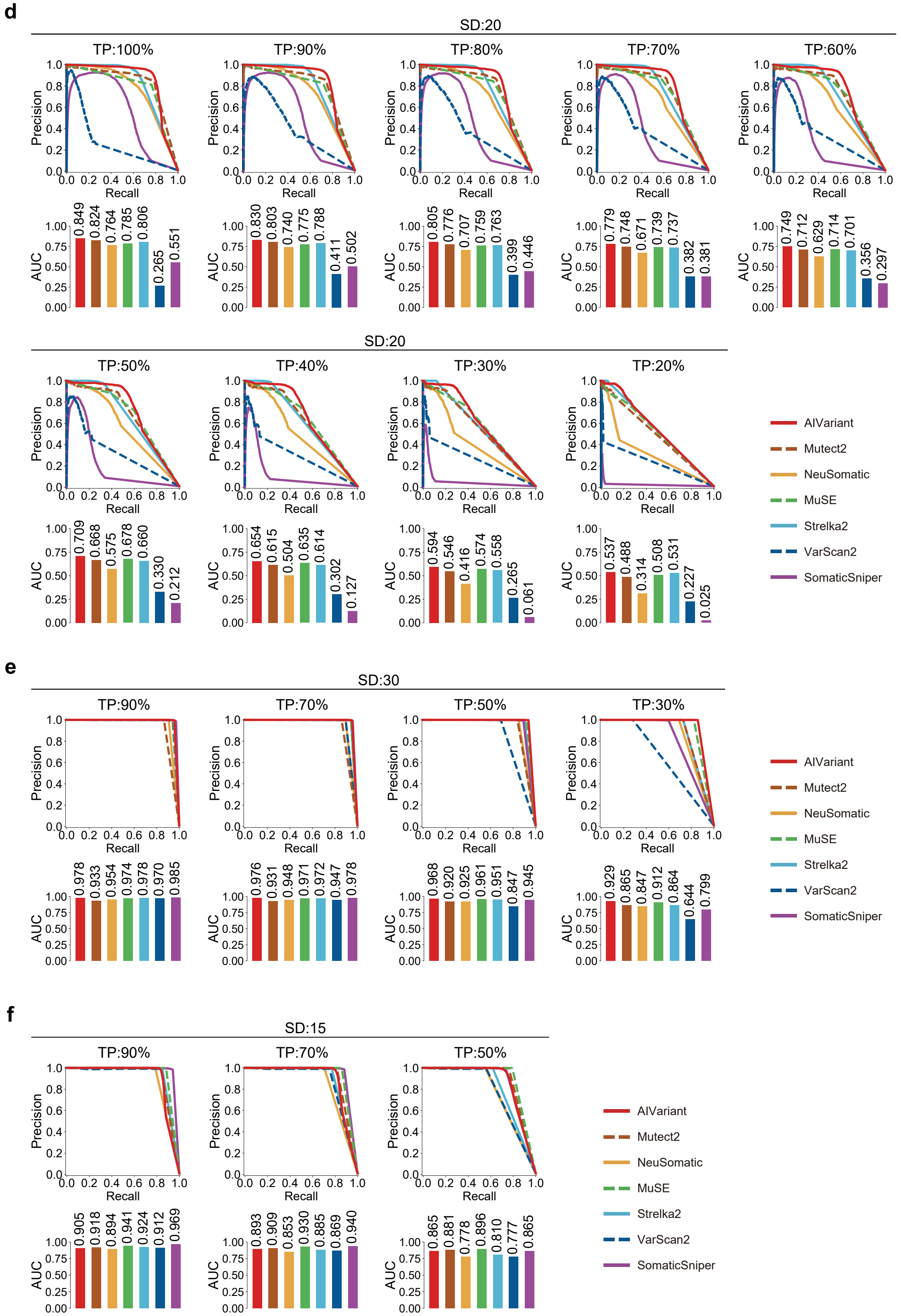
d
SD:20
TP:100%
TP:90%
TP:80%
TP:70%
TP:60%
Precision
Recall
AUC
0.849 0.824 0.764 0.785 0.806 0.265 0.551
0.830 0.803 0.740 0.775 0.788 0.411 0.502
0.805 0.776 0.707 0.759 0.763 0.399 0.446
0.779 0.748 0.671 0.739 0.737 0.382 0.381
0.749 0.712 0.629 0.714 0.701 0.356 0.297
SD:20
TP:50%
TP:40%
TP:30%
TP:20%
0.709 0.668 0.575 0.678 0.660 0.330 0.212
0.654 0.615 0.504 0.635 0.614 0.302 0.127
0.594 0.546 0.416 0.574 0.558 0.265 0.061
0.537 0.488 0.314 0.508 0.531 0.227 0.025
AIVariant
Mutect2
NeuSomatic
MuSE
Strelka2
VarScan2
SomaticSniper
e
SD:30
TP:90%
TP:70%
TP:50%
TP:30%
0.978 0.933 0.954 0.974 0.978 0.970 0.985
0.976 0.931 0.948 0.971 0.972 0.947 0.978
0.968 0.920 0.925 0.961 0.951 0.847 0.945
0.929 0.865 0.847 0.912 0.864 0.644 0.799
f
SD:15
TP:90%
TP:70%
TP:50%
0.905 0.918 0.894 0.941 0.924 0.912 0.969
0.893 0.909 0.853 0.930 0.885 0.869 0.940
0.865 0.881 0.778 0.896 0.810 0.777 0.865

g

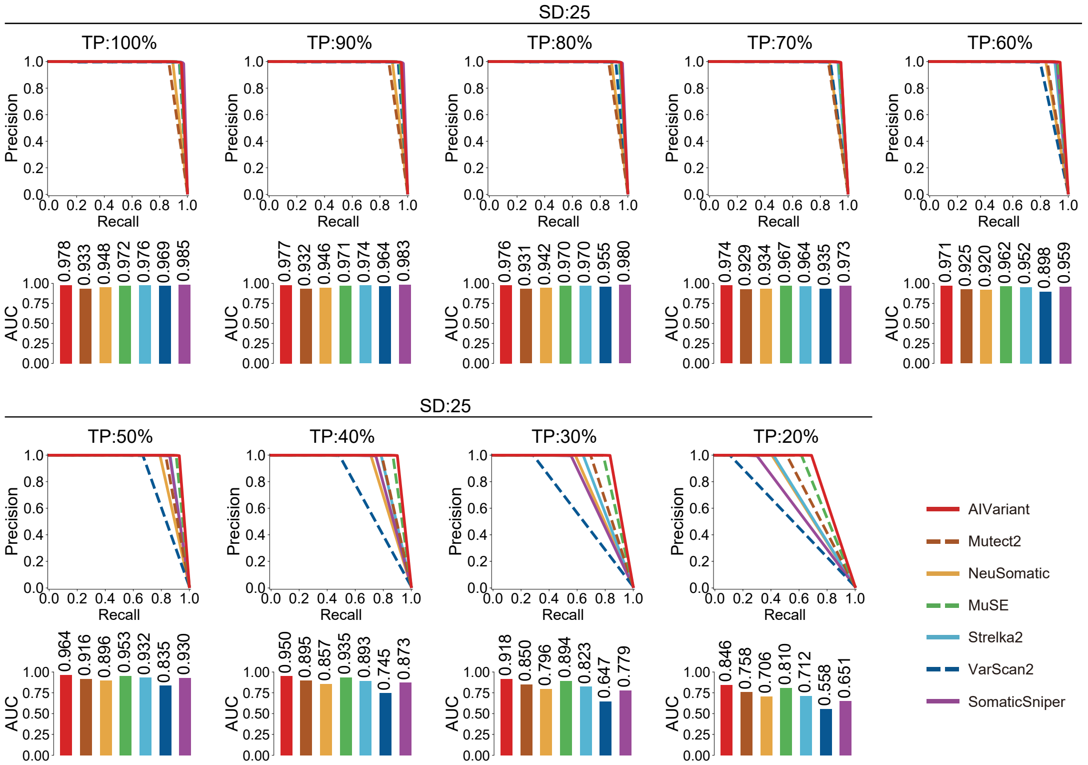
SD:25
TP:100%
TP:90%
TP:80%
TP:70%
TP:60%
Precision
Recall
AUC
0.978 0.933 0.948 0.972 0.976 0.969 0.985
0.977 0.932 0.946 0.971 0.974 0.964 0.983
0.976 0.931 0.942 0.970 0.970 0.955 0.980
0.974 0.929 0.934 0.967 0.964 0.935 0.973
0.971 0.925 0.920 0.962 0.952 0.898 0.959
SD:25
TP:50%
TP:40%
TP:30%
TP:20%
0.964 0.916 0.896 0.953 0.932 0.835 0.930
0.950 0.895 0.857 0.935 0.893 0.745 0.873
0.918 0.850 0.796 0.894 0.823 0.647 0.779
0.846 0.758 0.706 0.810 0.712 0.558 0.651
AIVariant
Mutect2
NeuSomatic
MuSE
Strelka2
VarScan2
SomaticSniper

h

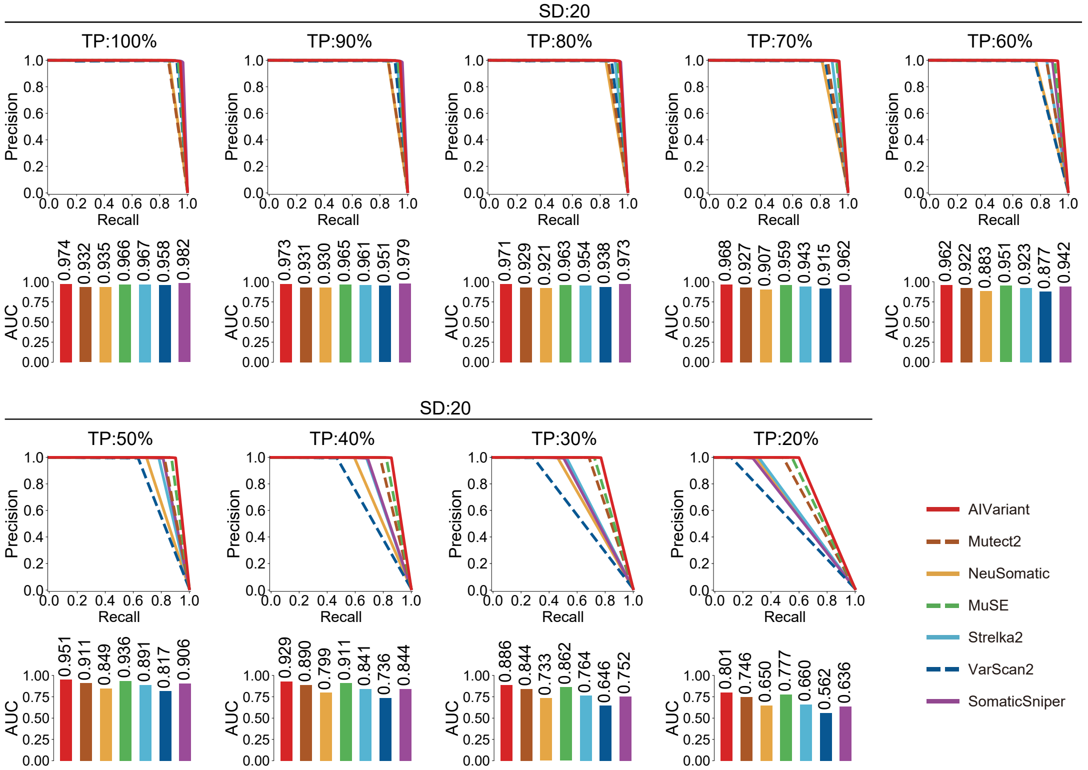
SD:20
TP:100%
TP:90%
TP:80%
TP:70%
TP:60%
Precision
Recall
AUC
0.974 0.932 0.935 0.966 0.967 0.958 0.982
0.973 0.931 0.930 0.965 0.961 0.951 0.979
0.971 0.929 0.921 0.963 0.954 0.938 0.973
0.968 0.927 0.907 0.959 0.943 0.915 0.962
0.962 0.922 0.883 0.951 0.923 0.877 0.942
SD:20
TP:50%
TP:40%
TP:30%
TP:20%
0.951 0.911 0.849 0.936 0.891 0.817 0.906
0.929 0.890 0.799 0.911 0.841 0.736 0.844
0.886 0.844 0.733 0.862 0.764 0.646 0.752
0.801 0.746 0.650 0.777 0.660 0.562 0.636
AIVariant
Mutect2
NeuSomatic
MuSE
Strelka2
VarScan2
SomaticSniper

Supplementary Fig. 5

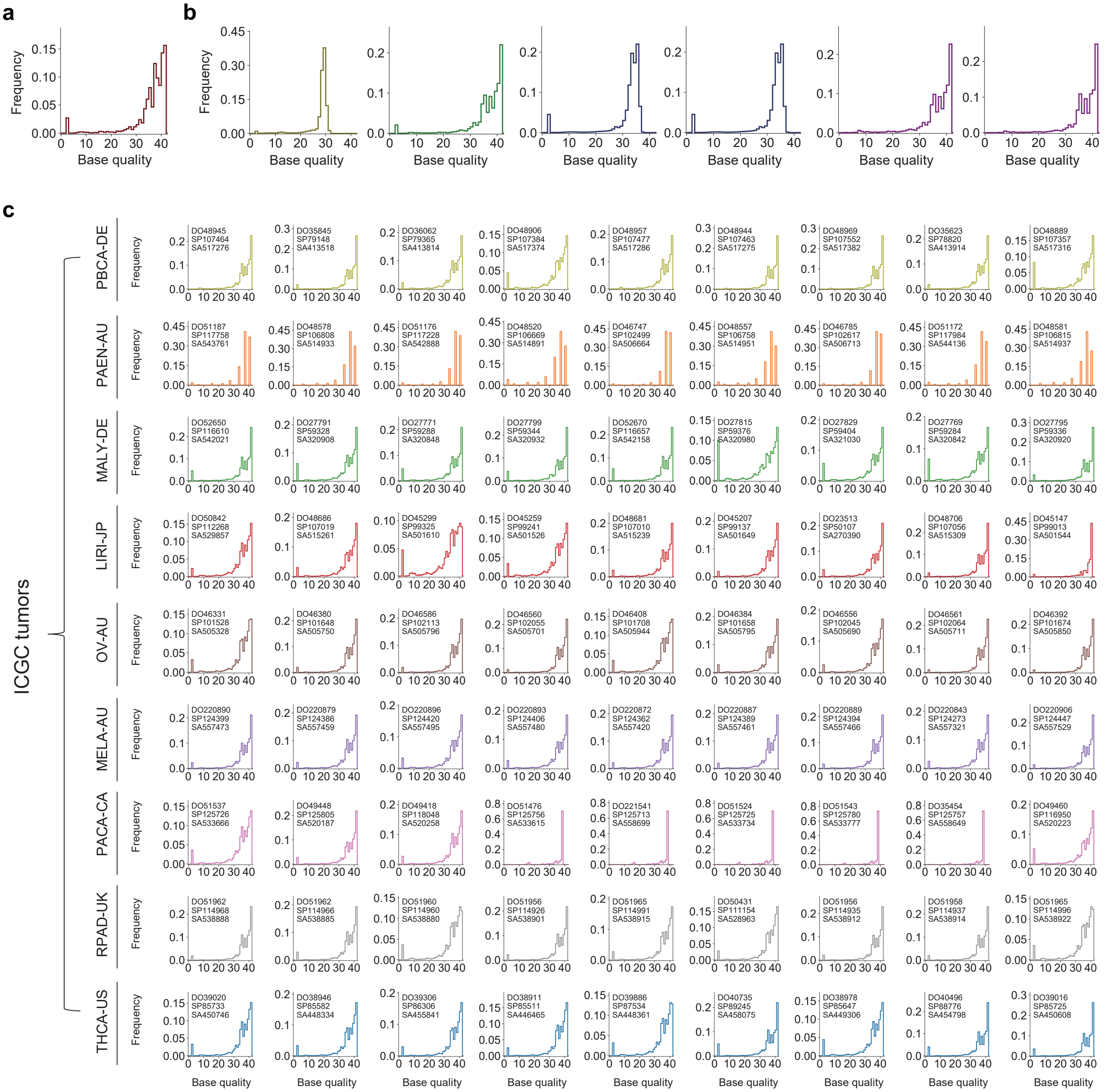

References

1. Bernstein, B.E. *et al.* The NIH Roadmap Epigenomics Mapping Consortium. *Nat. Biotechnol.* **28**, 1045-8 (2010).
2. Schuster-Böckler, B. & Lehner, B. Chromatin organization is a major influence on regional mutation rates in human cancer cells. *Nature* **488**, 504-7 (2012).
3. Cibulskis, K. *et al.* Sensitive detection of somatic point mutations in impure and heterogeneous cancer samples. *Nat. Biotechnol.* **31**, 213-9 (2013).
4. Alexandrov, L.B. *et al.* Signatures of mutational processes in human cancer. *Nature* **500**, 415-21 (2013).
5. Li, H. *et al.* A synthetic-diploid benchmark for accurate variant-calling evaluation. *Nat. Methods* **15**, 595-597 (2018).
6. Eberle, M.A. *et al.* A reference data set of 5.4 million phased human variants validated by genetic inheritance from sequencing a three-generation 17-member pedigree. *Genome Res.* **27**, 157-164 (2017).
7. Ewing, A.D. *et al.* Combining tumor genome simulation with crowdsourcing to benchmark somatic single-nucleotide-variant detection. *Nat. Methods* **12**, 623-30 (2015).
8. Consortium, I.T.P.-C.A.o.W.G. Pan-cancer analysis of whole genomes. *Nature* **578**, 82-93 (2020).
